# Pharmacological Evaluation of *Artemisia cina* Crude CO_2_ Subcritical Extract after the Removal of Santonin by Means of High Speed Countercurrent Chromatography

**DOI:** 10.3390/molecules25122728

**Published:** 2020-06-12

**Authors:** Zuriyadda Sakipova, Thais Biondino Sardella Giorno, Tolkyn Bekezhanova, Nikki Siu Hai Wong, Alma Shukirbekova, Patricia Dias Fernandes, Fabio Boylan

**Affiliations:** 1 School of Pharmacy, Kazakh National Medical University, Almaty 050000, Kazakhstan; sakipova.zb@gmail.com (Z.S.); tolkyn1984@mail.ru (T.B.); 2 Institute of Biomedical Sciences, Federal University of Rio de Janeiro, 21941-902 Rio de Janeiro, Brazil; thais.sardella.farma@hotmail.com (T.B.S.G.); patricia.dias.icbufrj@gmail.com (P.D.F.); 3 School of Pharmacy and Pharmaceutical Sciences and Trinity Biomedical Sciences Institute, Trinity College Dublin, Dublin 02, Ireland; wongn@tcd.ie; 4 Astana Medical University, NJSC Department of Pharmaceutical Disciplines, Nur Sultan 010000, Kazakhstan; shukiberkova.alma@gmail.com

**Keywords:** *Artemisia cina*, HSCCC, santonin-free CO_2_ subcritical extract, pectolinarigenin, antinociceptive, anti-inflammatory

## Abstract

*Artemisia* species are highly important due to their economic significance as medicines, fodder and food. *Artemisia cina* is an endemic species to Kazakhstan. In folk medicine, water extract of *A. cina* was used in the treatment of bronchial asthma while the alcohol extract has larvicidal and antituberculosis activity. The most common and most extensively studied compound from this species is the terpenoid santonin. The toxicity of this compound occurs at the doses of 60 mg for children and 200 mg for adults causing among other issues xanthopsia, leading to blindness. Having this in mind, the main idea of this work was to remove santonin from the crude extract and to check if the santonin-free extract would still be of any pharmacological importance. A CO_2_ subcritical extract was chromatographed using high-speed countercurrent chromatography (HSCCC) for the removal of santonin. The santonin-free CO_2_ subcritical extract (SFCO_2_E) as well as the isolated compound pectolinarigenin, a flavonoid, were assessed for their pharmacological actions. From the results obtained we can safely suggest that HSCCC is an efficient methodology to completely remove santonin from the CO_2_ subcritical extract. It was also possible to observe promising antinociceptive and anti–inflammatory activities for both SFCO_2_E and pectolinarigenin at concentrations that can justify the production of a phytomedicine with this endemic plant from Kazakhstan.

## 1. Introduction

Artemisia species are highly important due to their economic significance as medicines, fodder, and food. Examples of some biologically active substances isolated from plants belonging to the genus *Artemisia* are artemisinin, guaianolide, capillarisin, fisetin, quercetin, barrelin, artemalin, and barrelierin [1].

The valuable species of this genus have been, and some still are, commonly used in traditional medicines since ancient times to treat a wide range of ailments such as malaria, cancer, inflammation, and infections by fungi, bacteria, and viruses [1,2,3,4].

*Artemisia cina*, a shrubby aromatic plant commonly known as Levant wormwood or santonica, is native to the east of the Caspian Sea, in Afghanistan and in the Southern Ural region, with a preference for saline sandy soils. It is also an endemic species to Kazakhstan, in semi-desert areas where there are extremes of both high and low temperatures [5].

In folk medicine, a water extract of *A. cina* was used in the treatment of bronchial asthma [6]. The alcohol extracts have larvicidal [7] and antituberculosis activities [8]. The non-volatile compounds found in this species include santonin [5,9], artemisinin [5,10], monogynin, mibulactone, pseudosantonin and deoxypseudosantonin [5]. One to two percent of the crude drug is composed of a cineole-based essential oil [11] and this can be found within the unexpanded flower heads containing compounds such as terpineol, pinene, terpinene [5], α-terpineol, carvacrol, and several monoterpenes and sesquiterpene hydrocarbons [11].

The most common and most extensively studied compound from this species is the terpenoid santonin. This compound has shown powerful anthelmintic activities [5,12], which was first discovered in 1830 independently by both Kahler and Alm [13].

Other pharmacological activities have also been identified such as anti-fungal against several fungi [14] and significant anti-inflammatory, antipyretic [1,15] and analgesic activities [16]. However, santonin is no longer in use due to its potential toxicity [5,12,17,18]. It is known that santonin is toxic at the doses of 60 mg for children and 200 mg for adults [19]. A well-known side effect of excessive santonin intake is xanthopsia, a visual distortion where bright objects are perceived as yellow and sometimes dark surfaces have a violet appearance and in some serious cases, blindness can result [20,21]. The toxicity of this compound does not occur only from overdosing, but also when it has been administered over an extended length of time as the elimination of santonin from the body happens slowly, therefore acting as a cumulative poison [5].

Having this in mind, the main idea of this work was to remove santonin from this plant crude extract and to check if the extract would still be of any pharmacological importance. To achieve this aim, a methodology involving countercurrent chromatography was developed and this methodology also allowed for the isolation of other compounds, from which pectolinarigenin, a flavonoid, was pharmacologically evaluated alongside the crude extract.

## 2. Materials and Methods

### 2.1. Reagents and Materials

The solvents hexane, ethyl acetate, methanol, butanol and acetic acid were purchased from Trinity College Dublin HMF facilities (Dublin, Ireland). High-performance liquid chromatography (HPLC) grade water was obtained from a deionized water treatment system from PureLab Option, -Evoqua (Pittsburgh, PA, USA). Morphine, acetylsalycilic acid (ASA), formalin and carrageenan were purchased from Sigma-Aldrich, Dublin, Ireland.

### 2.2. Plant Material and Extraction

*A*. *cina* was collected on August 15, 2015 in the village Dermene, in the South-Kazakhstan region. Michael Petrov from the Institute of Botany (Botanic Garden of Almaty) was responsible for the identification of the collected plant material. *A*. *cina* leaves were first dried, ground to a powder and then extracted on a UUPE 5L subcritical CO_2_ (SC-CO_2_) extractor (KRI, Krasnodar, Russia) at Phyto-aroma, Kazakhstan. The extraction time was around 18 h with a pressure of 66 to 68 atm and temperature varying from 16 to 18 °C, yielding 2.6% of extract.

### 2.3. High Speed Countercurrent Chromatography (HSCCC) Separation Procedure

The High Speed Countercurrent Chromatography (HSCCC) was performed using an instrument IntroPrepTM-Quattro (AECS-QuikPrep, Cornwall, UK). The apparatus works by the action of centrifuge force in a rotation speed of 200 g. The column consists of a coil of polytetrafluoretylene (PTFE) tubing wrapped around a bobbin (internal diameter of the tube 2.0 mm, total volume 136 mL). A manual sample loop with a volume of 6 mL was used for this study.

### 2.4. Thin Layer Chromatography and Choice of HSCCC System

Analysis of *A. cina* aerial parts CO_2_ subcritical extract, solvent systems and fractions obtained after each HSCCC were performed by means of Thin Layer Chromatography using silica gel TLC plates 60F_254_ (Merck Art. check, Darmstadt, Germany). The mobile phase used for each TLC was composed of chloroform, methanol and water at 9:1:1 ratio. Visualization of the plates was possible by spraying the plate with H_2_SO_4_ 10% in ethanol followed by heating on a hot plate at 110 °C.

Four solvent systems (SS) composed of HEMWat (Hexane: ethyl acetate: methanol: water) at the proportions of 1:1:1:1; 1:2:1:2; 1:3:1:3 and 1:4:1:4 were tested to choose the best separation for the subcritical CO_2_ extract prepared from the aerial parts of *A. cina*. The solvent system (SS) utilized for this study was chosen according to the partitioning of the constituents of the plant material in both the upper and lower phases of the biphasic solvent system.

### 2.5. Preparation of Santonin-Free Extract (SFCO_2_E)

The solvent system chosen for the separation of *A. cina* aerial parts constituents was HEMWat 1:1:1:1. Solvent system was prepared in a separation funnel at room temperature. After the separation of the phases, both lower phase and upper phase were degassed by sonication for 5 min.

The chromatographic separation was performed using the normal phase mode in a tail to head orientation. The countercurrent chromatography (CCC) column was filled completely with the lower phase (aqueous phase) with a flow rate of 10 mL/min. When the column was filled up with the stationary phase (lower aqueous phase), rotation was switched on (200 g) and the mobile phase (upper organic phase) started to be pumped with a flow rate of 2 mL/min. After reaching phase equilibrium with 82% retention of the stationary phase, the sample was injected. Fractions of 4 mL were collected (2 mL/min) for a total of 50 test tubes (elution) and 30 tubes (extrusion).

Santonin appeared in fractions 47–55. These fractions were removed to produce the santonin-free extract (SFCO_2_E). Santonin was isolated, purified and after the confirmation of its structure by NMR, it was quantified in *A. cina* as well as in seven other species of *Artemisia*, using a new developed HPLC method [22].

### 2.6. Purification of Isolated Compound

After successive cycles of HSCCC using SFCO_2_E, the collected samples were grouped into 11 fractions according to the similarity of elution pattern and retention factor (Rf) observed on the TLC plates. HEMWat 1:1:1:1 was used as the solvent system again as described above. Fractions 15–29 showed the presence of yellow bands from which pectolinarigenin was isolated. Fractions 15–29 were put together and submitted to Gel-filtration Chromatography using a Sephadex LH-20 column obtained from Sigma-Aldrich (Dublin, Ireland) and eluted with methanol at an average flow rate of 0.5 mL/min. A complete purification was achieved allowing for the isolation of Pectolinarigenin at 98% purity.

### 2.7. NMR Identification

Structural elucidation of the isolated molecules was performed using an Agilent Technology (Santa Clara, CA, USA) 400NMR apparatus. Hydrogen and carbon 13 spectra were recorded on a BRUKER TOPSPIN 2.1 (1H-NMR: 400 and 600 MHz and 13C-NMR: 125 MHz) NMR spectrometry system using the Bruker pulse sequence standard (Bruker, Billerica, MA, USA). Two-dimensional measurements (homonuclear correlation spectroscopy (H-H COSY), Heteronuclear Multiple Bond Correlation (HMBC), Heteronuclear Multiple Quantum Coherence (HMQC)) were obtained on the same instrument with the usual pulse sequences. Peaks were visualized from ACD/NMR Processor Academic Edition software version 12.01.

Both SFCO_2_E and pectolinarigenin, an isolated flavonoid, were used to evaluate the pharmacological activities. Pectolinarigenin was isolated by a combination of one run of the HSCCC and size exclusion chromatography (Sephadex LH-20).

### 2.8. Animals

Male and female Swiss *Webster* mice (25–40 g, 8–10 weeks) with food and water ad libitum regimen for 24 h were kept in a controlled temperature environment (22 ± 2 °C, 60–80% humidity) and used in all in vivo experiments (groups of five to seven animals). The National Council for Control of Animal Experimentation (CONCEA), Biomedical Science Institute/UFRJ and Ethical Committee for Animal Research, approved the protocol for the animal experiments under the number DFBCICB015–04/16.

### 2.9. Antinociceptive and Anti-Inflammatory Activities In Vivo

Stock solutions of 100 mg/mL of the dried extract, pectolinarigenin and positive controls (ASA, morphine and dexamethasone) were prepared by dissolving in DMSO and kept at −20 °C.

#### 2.9.1. Formalin-Induced Licking Response

Acute nociception was induced through an injection of 20 µL of formalin (2.5% *v*/*v*) into the dorsal surface of the left hind paw of mice according to the method described by Matheus et al. [23]. The recording of time animals were licking their paw was during two phases: the first phase (neurogenic pain response) until 5 min after injection and the second phase (inflammatory pain response) between 15 to 30 min post-injection. Pre-treatment with intraperitoneal doses of SFCO_2_E (10, 30, and 100 mg/kg), pectolinarigenin (1, 3, and 10 mg/kg), ASA (200 mg/kg), morphine (2.5 mg/kg) or vehicle (ultrapure water) 30 min before formalin injection was performed in the studied animals.

#### 2.9.2. Thermal-Induced Nociception Model Using a Hot Plate

The method described by Sahley and Bernston [24] was used to study animals for this test. Animals were placed on a hot plate (Insight Equipment, São Paulo, Brazil) set at 55 ± 1 °C. Jumping, licking or lifting the back paw (reaction times) were recorded at 30, 60, 90, 120, 150, and 180 min after intraperitoneal administration of SFCO_2_E (10, 30, and 100 mg/kg), morphine (2.5 mg/kg) or vehicle (ultrapure water). The mean of the reaction time 60 and 30 min before treatments were considered baseline—defined as the normal reaction of the animal to the temperature. The increase in the baseline (%) calculated by the formula: (reaction time × 100/baseline—100) was quantified as antinociception.

#### 2.9.3. Capsaicin-Induced Nociception

The protocol used to evaluate the capsaicin-induced nociception was described by Sakurada et al., 1998 [25] with a few modifications. Capsaicin solution (20 μL/paw, 1.6 μg/paw) was injected by intraplantar route in the right hind paw of mice. After capsaicin injection, the animals were immediately placed in a transparent box (separate from each other) and the licking-time or biting of the capsaicin-injected paw was recorded for a period of time (5 min) and considered as the nociceptive reaction. Animals were pretreated 60 min prior to the intraplantar injections with either pectolinarigenin (1 mg/kg) or vehicle by oral gavage.

#### 2.9.4. Glutamate-Induced Nociception

The protocol used to evaluate the glutamate-induced nociception was described by Beirith et al., 2002 [26] with a few modifications. Glutamate solution (3.7 ng/paw) was injected under the surface of the right hind paw of mice 60 min after treatment with pectolinarigenin (1 mg/kg) or vehicle by oral gavage. After glutamate injection, the animals were immediately placed in a transparent box (separate from each other) and the licking-time or biting of the glutamate-injected paw was recorded for a period of time (15 min).

#### 2.9.5. Carrageenan-Induced Cell Migration Using the Subcutaneous Air Pouch (SAP) Model

Two consecutive administrations of sterile air (8 and 10 mL, respectively) in the intrascapular area of the mice with intervals of four days confectioned and maintained the air pouches. Animals received sterile carrageenan suspension (1%—1 mL) or sterile saline (1 mL) in the SAP two days after the last injection. Mice were pre-treated intraperitoneally with 10, 30, or 100 mg/kg of SFCO_2_E, 1, 3, or 10 mg/Kg with pectolinarigenin or dexamethasone (2.5 mg/kg) 30 min before the carrageenan administration. Twenty-four hours after carrageenan injection, animals were euthanised and the cavity was washed with 1 mL of sterile saline. The determination of the total number of cells was performed by the collection of aliquots of the exudates together with a blood sample and a bone narrow wash (1 mL sterile saline in femur). The exudates were centrifuged at 1200 rpm for 10 min at 4 °C, and the supernatants were collected and stored at −20 °C until use based on the work by Raymundo et al. [27].

### 2.10. Statistical Analysis

In vivo experimental groups were composed of five to seven animals. Results are expressed as the mean ± standard deviation (S.D.) for the formalin-induced liking response, subcutaneous air pouch, capsaicin- and glutamate-induced nociception models. The hot plate results were quantified as area under curve, expressed as mean ± standard error (SEM) compared to control. Statistical significance was calculated by analysis of variance (ANOVA) followed by Bonferroni test and student-t test in the case of capsaicin- and glutamate-induced nociception. *P* values less than 0.05 (* *p* < 0.05) and 0.01 (** *p* < 0.01) were considered significant.

## 3. Results

### 3.1. Phytochemistry

#### 3.1.1. Isolation of the Pectolinarigenin

HEMWat (Hexane: ethyl acetate: methanol: water) in a ratio of 1:1:1:1 presented the best distribution of the compounds of interest in the lower or aqueous phase and upper or organic phase when analysed on a TLC plate using chloroform: methanol: water in a ratio of 9:1:1, as mobile phase. Initially, 80 tubes of SFCO_2_E from the aerial parts of *Artemisia cina* were collected from the HSCCC using HEMWat as a solvent system, as mentioned before. The tubes were joined into fractions accordingly the chromatographic similarities of the constituents (Rf value observed on the TLC plates). A compound (Rf~0.51) was isolated and purified by means of gel filtration chromatography.

#### 3.1.2. Structural Elucidation

**Figure 1:**
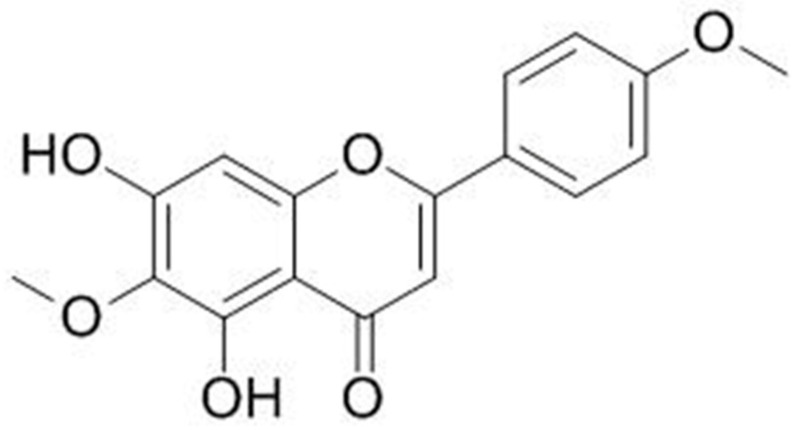
Pectolinarigenin isolated from *Artemisia cina* CO_2_ subcritical extract.

The isolated compound had its structure established as pectolinarigenin (Figure 1) by means of ^1^H-NMR and ^13^C-NMR and comparison to the literature (Table 1). The chemical signals obtained for the isolated substances are described (in ppm) as follows:

Pectolinarigenin, yellow precipitate; Rf 0.51 (chloroform: methanol; 9:1); ESI-MS for C_17_H_14_O_6_ *m*/*z*; 314.3 [M + H]^+^; 1H-NMR (600MHz, CDCl3) δH: 3.91 (3H, *s*, MeO); 4.03 (3H, *s*, MeO); 6.61 (1H, *s*, H-3); 6.59 (1H, *s*, H-8); 7.03 (1H, *d*, H-3′ and H-5′); 7.86 (1H, *d*, H-2′ and H-6′); 13.05 (1H, *s*, OH-5); δC (150MHz, CDCl3): 55.56 (C-4′); 60.87 (MeO-6); 93.64 (C-8); 103.66 (C-3); 105.6 (C-4a); 114.5 (C-3′ and C-5′); 123.52 (C-1′); 128.09 (C-2′ and C-6′); 130.62 (C-6); 152.33 (C-8a); 153.2 (C-5); 155.46 (C-7); 162.61 (C-4′); 164.21 (C-2); 182.98 (C-4). These data are in agreement with that reported by Hase et al., 1995 [28] and Segueni et al., 2016 [29].

### 3.2. Pharmacology

#### 3.2.1. Nociception-Induced Model Using Formalin

In this experiment, the treated groups were compared with their corresponding controls, such as, blank (vehicle), ASA or morphine. All the data collected were analyzed using one-way ANOVA, * *p* < 0.05 followed by the Bonferroni test.

**Figure 2:**
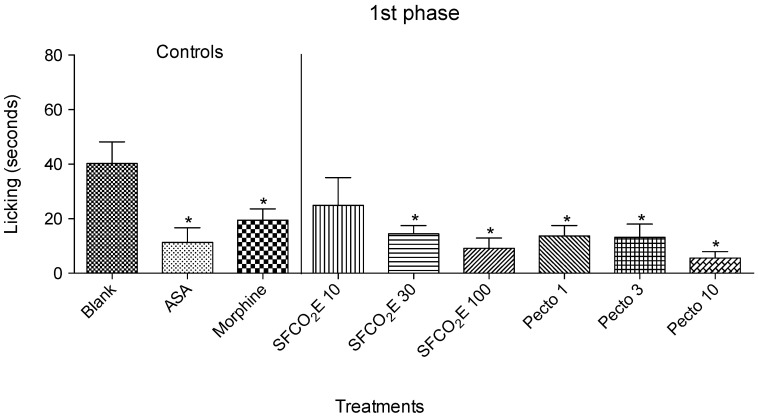
The results from the first phase of formalin of treated mice (santonin-free CO2 subcritical extract (SFCO_2_E) at 10, 30, and 100 mg/kg or pectolinarigenin (Pecto) at 1, 3, and 10 mg/kg) were compared with mice that were given vehicle (blank), ASA (200 mg/kg) or morphine (2.5 mg/kg) used as positive controls. This figure shows the decrease of the bar against the untreated mice. The treatments which show a significant decrease in the bar size compared to blank are marked with (*), *p* < 0.05.

Therefore, in the first phase, there is a significant reduction in licking time for the SFCO_2_E group at the two highest concentrations tested (30 and 100 mg/kg) and for pectolinarigenin also at the three concentrations tested (1, 3, and 10 mg/kg). The positive controls used were morphine and ASA. (Figure 2).

**Figure 3:**
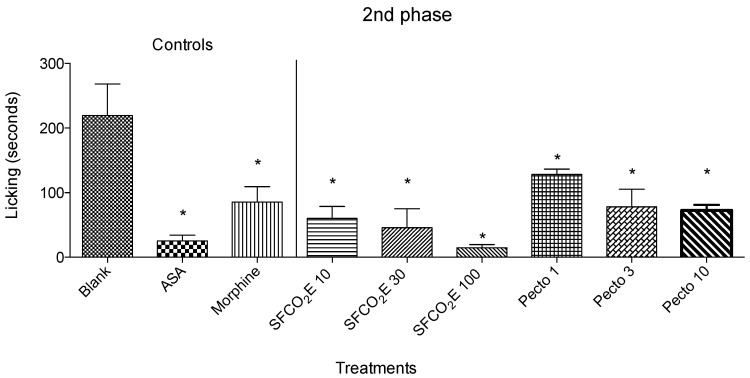
The results from the second phase of formalin of treated mice (SFCO_2_E at 10, 30, and 100 mg/kg or pectolinarigenin at 1, 3, and 10 mg/kg) were compared with mice that were given vehicle (blank), ASA (200 mg/kg) and morphine (2.5 mg/kg) used as positive controls. This figure shows the decrease of the bar against the untreated mice. The treatments which show a significant decrease in the bar size compared to blank are marked with (*), *p* < 0.05.

In the second phase of the formalin test, all SFCO_2_E concentrations tested (10, 30, and 100 mg/kg) and all pectolinarigenin concentrations tested (1, 3, and 10 mg/kg) showed a significant reduction in the licking time (Figure 3).

SFCO_2_E showed a more significant reduction in licking time in the second phase than in the first phase, whereas the mice treated with 1 and 3 mg/kg of pectolinarigenin showed a greater significance in the first phase compared to the second phase.

#### 3.2.2. Capsaicin-Induced Nociception

**Figure 4:**
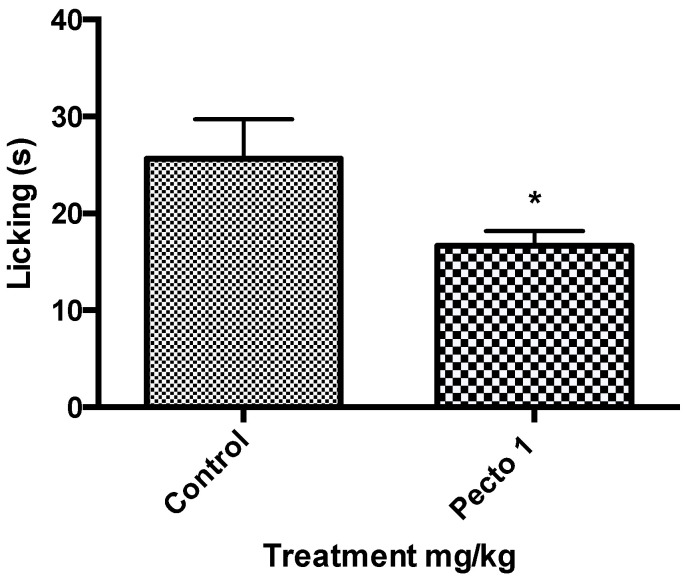
The preliminary results of Pecto 1 (1 mg/kg of pectolinarigenin) using students t-test to analyse the data (* *p* < 0.05).

Figure 4 shows the results of the 1 mg/kg pectolinarigenin treatment given to mice (*n* = 6) and these were compared with the control (no treatment) in a capsaicin-induced nociception experiment. The asterisk (*) indicates there to be a significant reduction of licking when the mice were treated with 1 mg/kg of pectolinarigenin.

#### 3.2.3. Glutamate-Induced Nociception

**Figure 5:**
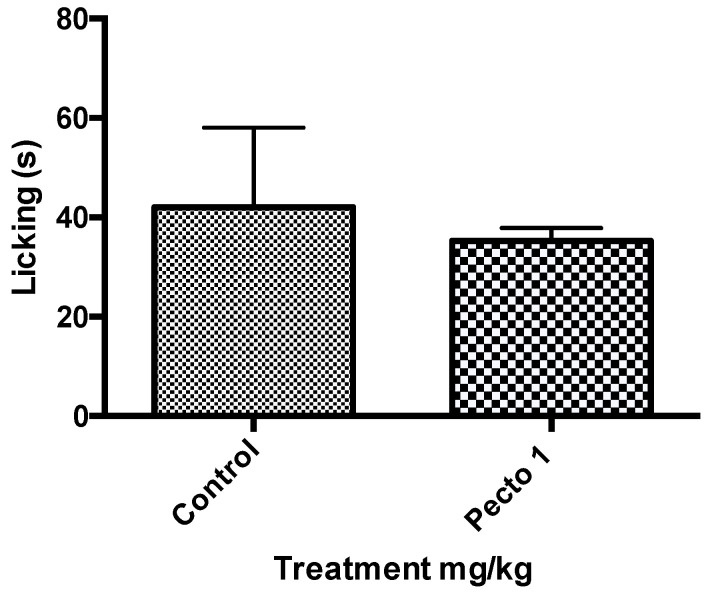
The pilot results of 1 (P) (1 mg/kg of pectolinarigenin) using students t-test to analyse the data. Treatment with Pectolinarigenin was not statistically significant.

In Figure 5, pectolinarigenin was tested on mice (*n* = 6) and the results were compared with the control (no treatment) in a glutamate-induced nociception experiment. There were no significant reductions in the licking time of the mice treated in this test.

#### 3.2.4. Thermal-Induced Nociception Model Using a Hot Plate

**Figure 6:**
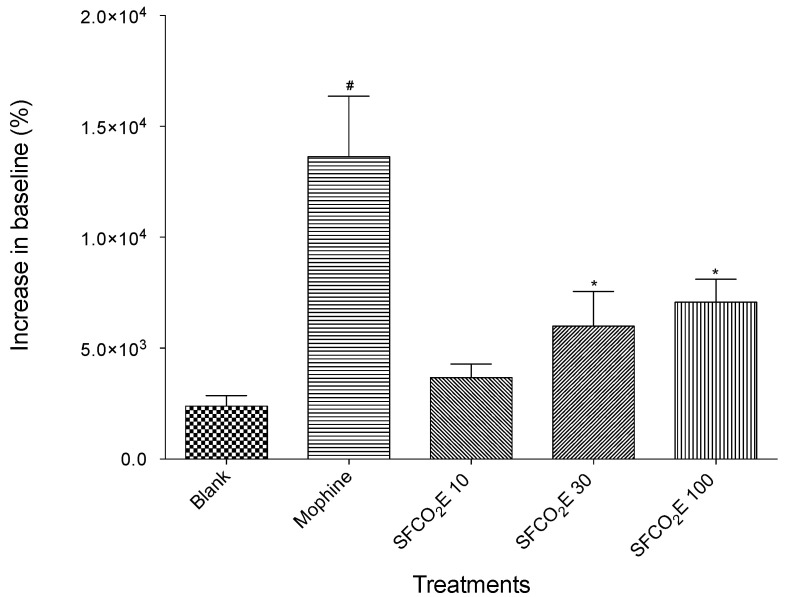
The results from the hot plate model of treated mice (SFCO_2_E at 10, 30, and 100 mg/kg) were compared with mice that were given vehicle (blank) and morphine (2.5 mg/kg) used as positive control. This figure shows the increase of the baseline against the untreated mice (blank). The treatments which show a significant increase in the baseline size compared to blank are marked with (*), *p* < 0.05. (#), *p* < 0.01.

The results from the heat-induced nociception model can be seen in Figure 6 showing the SFCO_2_E at doses of 10, 30, and 100 mg/kg, respectively, a blank control (no treatment), and a positive control, morphine. A statistically significant increase in the baseline was seen for SFCO_2_E at the two highest concentrations (30 and 100 mg/kg). Unfortunately, the quantity of pectolinarigenin available was not enough to test in this experiment. All the data collected were analyzed using one-way ANOVA, R^2^ > 0.95 followed by the Bonferroni test.

#### 3.2.5. Carrageenan-Induced Cell Migration Using Subcutaneous Air Pouch (SAP) Model

**Figure 7:**
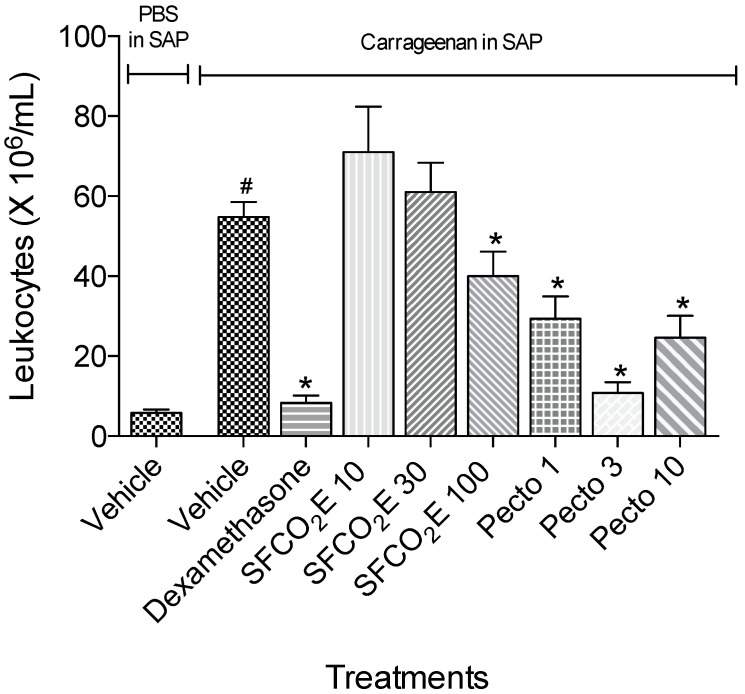
The results from the total leukocytes migration to the air pouch of treated mice (SFCO_2_E at 10, 30, and 100 mg/kg, pectolinarigenin at 1, 3, and 10 mg/kg or dexamethasone at 2.5 mg/kg were compared with mice that were given vehicle by oral gavage 60 min before carrageenan (1%, 1 mL) or PBS (1 mL) injection into subcutaneous sir pouch (SAP). This figure shows the decrease of the bar against the carrageenan injected group that received vehicle (p.o). The treatments that show a significant decrease in the bar size compared to the group receiving carrageenan are marked with (*), *p* < 0.05. (#), *p* < 0.01.

In this model, total leukocytes were analyzed from the subcutaneous air pouch exudates (Figure 7), from five treatment groups of mice (*n* = 7). The data collected here were analyzed using one-way ANOVA, * *p* < 0.05 followed by the Bonferroni test.

In this model, carrageenan was injected into the subcutaneous air pouch of all the treatment groups except for the phosphate-buffered saline (PBS) control group. As shown in Figure 7, carrageenan induces leukocyte migration into the air pouch. From the treated groups, it can be seen that only the concentration of 100 mg/kg of the SFCO_2_E and pectolinarigenin at the three tested concentrations (1, 3, and 10 mg/kg) were able to show a significant lowering in total leukocytes migration into the air pouch compared to the group receiving carrageenan and treated with vehicle by oral gavage.

## 4. Discussion

Although pectolinarigenin (Figure 1) is not a novel compound, no previous studies have isolated pectolinarigenin from *Artemisia cina*—therefore, this is the first time this flavonoid is being reported in this species. Table 1 shows the NMR Spectroscopic Data (600 MHz, CDCl3) for pectolinarigenin with ^1^H- and ^13^C-NMR values to compare from this study [28,29]. As reported by Ryakhovskaya and Sapko in 1985 [30], the hyroxy derivative hispidulin, has been previously isolated from *Artemisia cina*. Although in this study the isolation of hispidulin was not achieved, there is a high chance that it was present in the plant as there were two obvious yellow bands seen prior to pectolinarigenin purification.

*Artemisia cina* has been widely used in traditional medicines, for example, in the treatments of bronchial asthma [6], worms [7] and tuberculosis [8]. This species has been most extensively studied in regard to santonin because it is the primary constituent in the plant. Santonin was considered of interest to investigate as it is a sesquiterpene lactone, and these structures are known to be active constituents of the medicinal plants used in traditional medicine for the treatment of inflammatory diseases [31]. However, *Artemisia cina* has not yet been studied in regard to pain or inflammation, therefore, in this study pain and inflammation-induced models were used to examine the pharmacological properties of this plant. The SFCO_2_E and pectolinarigenin (P) were evaluated in pharmacological models for nociception and inflammation.

Pain is an unpleasant sensation produced by either damaged tissues or stimuli that are capable of damaging the tissue and these harmful stimuli are detected by periphery sensory nerve fibers called nociceptors [32,33]. These nerve fibers release neurotransmitters such as glutamate and neuropeptides, and these further activate second order neurons conveying information to the thalamus where the sensation of pain is processed [33]. Its functions have obvious physiological roles, such as warning the body of potentially dangerous stimuli or attracting attention to inflamed tissues [34].

Morphine was used as appositive control in these experiments as it induces an increase in ATP-sensitive K^+^ channel, followed by a hyperpolarization of the nociceptive neurons, restoring the normal high nociceptor threshold which results in an anti-nociceptive effect [33].

Formalin was used to induce nociception in the mice as it is known to induce biphasic activities in afferent fibers [35]. The first phase is short lasting, and the pain witnessed is a result of direct stimulation of nociceptors, activating the C-fiber causing a peripheral stimulus, which corresponds to neurogenic pain. The second phase, on the other hand, is the period of sensitization [36] where the peripheral nerve fibers are stimulated to induce pain [37]. The intensity of the behavior observed in mice are dependent on the concentration of formalin administered [36], and it was therefore important to note the concentration because if the concentration of formalin used is too high, then it may overpower and mask any analgesic effect of the drug being tested.

In this study there were three treated groups showing significant effects in the first phase, from the SFCO_2_E and pectolinarigenin indicating to have nociception mainly related with the peripheral mechanism. In the second phase, there were six groups, from the SFCO_2_E and pectolinarigenin, suggesting a potential anti-inflammatory effect. These results prove that there is a potential for this plant to be used in antinociception and anti-inflammation. Pectolinarigenin was isolated from the SFCO_2_E, and therefore it could potentially be one of the actives possessing the anti-nociceptive and anti-inflammatory properties in the extract. With these results, further investigations were carried out in attempt to analyze these activities in more detail.

After the investigation using the formalin test, preliminary investigations were carried out on pectolinarigenin as it showed to have a significant in the formalin test. Capsaicin is an active ingredient of the capsicum pepper, which gives the sensation of burning pain and it is also an excitatory neurotoxin [38] and acts on the vanilloid receptors (TRVP1) [39], which detect noxious heat [38,40]. The results from this test showed that pectolinarigenin significantly decreases the nociception of capsaicin, which suggests that it may act on the TRVP1 receptors, which mediate the release of several neurotransmitters.

The choice to study glutamate-induced nociception with the flavonoid was due to the fact that it is an excitatory amino acid, which mediates noxious stimuli. An increase in glutamate levels activate N-methyl-D-aspartic acid (NMDA) receptors which in turn increases intracellular levels of Ca^2+^, eventually resulting in the increased release of nitrate and nitric oxide amassing their concentration in the effected cells [33]. Nitric oxide (NO) is a major mediator of nociception in pain conditions [41] by acting on nerve-endings as a neurotransmitter [33]. Therefore, if glutamate were introduced externally, it would be expected to cause an increase in the nociception sensed by the mice, thus increasing the paw licking time (per second). The results from this test using pectolinarigenin show a small decrease but not a significant reduction in licking response compared to the control, which suggests that the antinociceptive activity of pectolinarigenin shown in the formalin test does not directly affect the neurotransmitter glutamate.

The hot plate test used a thermal stimulus to examine the nociceptive properties of SFCO_2_E, and showed a significant increase in the baseline at the concentrations of 30 and 100 mg/kg for the thermal stimuli suggesting that it has some antinociceptive properties at a central level. Further studies will be necessary to determine the mechanism for this nociceptive activity seen.

Inflammation is a reaction by the body as a form of protection and it is also a healing process for repairing injured tissues [42]. It is a highly complex, interacting, and redundant process [43,44].

Mast cells proximity to the external environment allows them to be the first response external harmful stimuli such as pathogens and allergen exposure. When mast cells are activated, they undergo degranulation, which stimulates the production and release of pre-formed soluble mediators for example proteases, proteoglycans, lysosomal enzymes, Tumor Necrosis Factor alpha (TNF-α), and others [45].

TNF-α is a cytokine involved in the inflammatory processes and is one of the chief stimulators of producing the acute-phase proteins. Acute phase proteins are proteins whose plasma concentration increases or decreases by at least 25% during inflammation and these changes reflect the manifestation and intensity of the inflammatory process [44].

Neutrophils are the most abundant leukocyte and play a vital role in stimulating host response. Their presence manages the active procedures of inflammation resolve to restore the cell function by clearing the tissue of any infiltrated leukocytes, therefore allowing tissue repair and regeneration [46]. Leukocytes store large amounts of glucocorticoid-induced protein annex A1 (AnxA1) and they are activated when there is an increase in intracellular Ca^2+^ concentrations. These proteins are secreted from neutrophils, which apoptosis at the site of inflammation and attract blood borne monocytes, eventually increasing the number of macrophages that are critical for the clearance of dead cells and cellular debris [46]. Interleukin- 1β (IL-1β) is another cytokine produced by macrophages after peripheral nerve injury and IL-1βcan upregulates other pro-nociceptive mediators and also directly affect nociceptors inducing signaling cascades that lead to the activation of other molecules [47] such as prostaglandins [48], which play an important role in inflammation, synergizing with other mediators to stimulate enhanced vascular permeability and edema [49] allowing more leukocytes into the area of inflammation.

Dexamethasone was used as a positive control in the tests where applicable, as it is an anti-inflammatory steroid with the ability to selectively inhibit cytokine or the endotoxin of cyclooxygenase-2 (COX-2) enzyme, which is induced by inflammatory cytokines [50].

The inhibitory results produced by the extract and pectolinarigenin treatments in the second phase of the formalin model led to the evaluation of its effect in the subcutaneous air pouch model, a model of acute inflammation. The injection of carrageenan (1%), an inflammatory agent, into the mice air pouches increases the total leukocytes that migrated to the cavity, upregulating the production of IL-1β and other inflammatory cytokines [33,47].

The results showed that pectolinarigenin possessed significant responses. Pre-treatment of animals at the doses of 1, 3, and 10 mg/kg were effective in reducing the total leukocytes present in the air pouch exudates with 3 mg/kg being the most active. This indicates that this flavonoid significantly suppressed cell migration.

Anti-inflammatory drugs are among the most widely used therapeutic agents in the world, but they have some limitations regarding their potency, effectiveness and adverse effects. NSAIDs (non-steroidal anti-inflammatory drugs) have adverse gastrointestinal effects and selective NSAIDs for cox-2 have been associated with minor but prominent cardiovascular changes in some patients [51]. Steroidal anti-inflammatory drugs have prominent adverse effects due to the common action of steroids. The recent modulators or anti-cytokines (anti IL-1β and anti-TNF-α) are very expensive and the route of administration is subcutaneous, at least twice a week, which decreases treatment compliance. Due to these inconveniences, it is necessary to search for natural substances with anti-inflammatory potential that can provide new drugs at low cost and with reduced adverse effects, or that at least can assist in the understanding of the complex mechanisms of action [52].

Literature data show that molecules of plant origin have important anti-inflammatory activities and that many of their actions are related to the ability to inhibit the synthesis or action of cytokines, chemokines and adhesion molecules, arachidonic acid and oxide nitric pathways, in addition to inhibition of NF-κB [53].

Plant preparations, in turn, often inhibit more than one course of action, maximizing the anti-inflammatory effects and minimizing the adverse reactions [54]. This is partially explained by the fact that there is a mixture of substances that could act synergistically or antagonistically at different receptors and pathways. In addition, it is suggested that products of plant origin have potential to be used in the form of mono preparations or in combination with current medications, with the aim of reducing costs and side effects and increasing efficiency [52].

## 5. Conclusions

This study has provided much useful information regarding the phytochemistry and pharmacological activity of *A. cina*. A quick one-step HSCCC method was used to produce the santonin-free extract used for further analysis.

Pectolinarigenin, a non-polar flavonoid, isolated from fractions obtained from the HSCCC analysis and further purified by size-exclusion, proved to be of pharmacological importance.

Results from the different methodologies allowed us to conclude that *A. cina* and pectolinarigenin possess potential antinociceptive and anti-inflammatory activities. CO_2_ subcritical extract seems to be active both in cases of peripheral and central nociception. On the other hand, pectolinarigenin is active for peripheral nociception in part through an action in the capsaicin receptors. Therefore, further studies with various antagonists would be necessary to confirm the possible mechanisms of action of the extract and pectolinarigenin.

Additionally, further studies using cytokines, chemokines, growth factors, reactive oxygen species (ROS), nitric oxide synthases (NOS) and prostaglandins are needed for the evaluation of the full anti-inflammatory potential of pectolinarigenin.

## Figures and Tables

**Table 1:** ^1^H- and ^13^C-NMR data of pectolinarigenin (600 MHz for ^1^H-NMR and 125 MHz for ^13^C-NMR, CDCl3). The data collected from published papers were used to confirm that the structure of the flavonoid isolated.

Position	Own Data		Hase et al., 1995 [28]		Segueni et al., 2016 [29]	
	δ_C_, mult.	δ_H_, (*J* in Hz)	δ_C_, mult.	δ_H_, (*J* in Hz)	δ_C_, mult.	δ_H_, (*J* in Hz)
2	164.2		163		163.3	
3	103.7	6.61, *s*	102.8	6.86, *s*	103.9	6.83
4	182.9		181.3		182.6	
4a	105.6		103.9		104.1	
5-OH	153.2	13.05, *s*	153.4	13.00, *s*	152.2	13.01
6	130.6		131.2		131.3	
7-OH	155.5		157		152.3	10.67
8	93.6	6.59, *s*	94	6.63, *s*	94.3	6.59
8a	152.3		152.1		151.9	
1′	123.5		122.7		123.7	
4′	162.6		162		160.9	
2′/6′	128.1	7.86, *d*	127.9	8.03, *d*	128.8	8.00
3′/5′	114.5	7.03, *d*	114.3	7.12, *d*	114.1	7.08
MeO-4′	55.5	3.91, *s*	55.3	3.77, *s*	55.8	3.75
MeO-6	60.8	4.03, *s*	59.6	3.86, *s*	60.4	3.87
